# Coronary changes and cardiac events in children diagnosed with kawasaki disease without initial coronary aneurysm**:** A multicenter retrospective cohort study

**DOI:** 10.3389/fped.2023.1121905

**Published:** 2023-03-15

**Authors:** Chanikarn Wongbundit, Chodchanok Vijarnsorn, Varisara Pornprasertchai, Pimonrat Somkittitham, Densiri Bositthipichet, Tikamporn Tongbunnum, Prakul Chanthong

**Affiliations:** ^1^ Division of Pediatric Cardiology, Department of Pediatrics, Faculty of Medicine, Siriraj Hospital, Mahidol University, Bangkok, Thailand; ^2^ Department of Pediatrics, Phrapokklao Hospital, Chanthaburi, Thailand; ^3^ Department of Pediatrics, Nakhon Pathom Hospital, Nakhon pathom, Thailand; ^4^ Department of Pediatrics, Chao Phraya Yommarat Hospital, Suphanburi, Thailand

**Keywords:** kawasaki disease (KD), coronary artery aneurysm (CAA), coronary ectasia, coronary artery *z* score, echocar diography, adverse cardiac events

## Abstract

**Background:**

Kawasaki disease (KD) is a systemic vasculitis affecting young children, which may lead to coronary artery aneurysm (CAA). The optimal timing of serial echocardiography in patients with uncomplicated KD is debated.

**Objectives:**

To assess changes in coronary artery Z-scores from the initial diagnosis, two weeks, eight weeks, and one year following diagnosis and adverse cardiac events in children diagnosed with KD without initial CAA.

**Methods:**

Retrospective chart reviews of four referral centers in Thailand were conducted of all children who were diagnosed with KD without initial CAA (coronary artery Z-score < 2.5) between 2017 and 2020. Eligibility criteria included the absence of congenital heart disease and patients with available echocardiographic evaluations at baseline and at eight weeks of illness. The two-week and one-year echocardiographies were reported. Adverse cardiac events at one year from diagnosis were explored. The primary outcome was a maximal coronary Z-score on the follow-up echocardiography at eight weeks and one year.

**Results:**

Of 200 patients diagnosed with KD, 144 patients (72%) did not have CAA. A total of 110 patients were included in the study. The median age was 23 months (IQR, 2–39 months) and 60% were male. Fifty patients (45.5%) had incomplete KD, and four (3.6%) received a second intravenous immunoglobulin treatment. Of 110 patients, 26 patients (23.6%) had coronary ectasia (Z-score of 2–2.49) on their initial echocardiographic examination. Sixty-four patients were evaluated in two-week echocardiographic studies, which showed four new small CAAs and five coronary ectasia. At 8 weeks, 110 patients had undergone complete echocardiographic studies. No patient had residual CAAs. Only one patient had persistent coronary ectasia that regressed to normal within one year. At one-year follow-up (*n* = 90), no cardiac events were reported.

**Conclusion:**

New CAA in-patients with KD who had no previous CAA in their initial echocardiography are rare. In addition, patients who had normal echocardiographic follow-up at two weeks or eight weeks mostly continued to be normal at one year. The optimal timing of the echocardiographic follow-up should be at two to eight weeks in patients without initial CAA, who still have a coronary artery Z-score < 2 at the second echocardiography.

**Trial registration**: TCTR20210603001.

## Introduction

Kawasaki disease (KD) is an acute febrile illness with inflammation of medium-sized vessels, often affecting young children. Coronary artery involvement is common and varied in its severity from transient, mild coronary artery dilation (so called coronary ectasia) to severe forms such as giant coronary artery aneurysm (CAA). The incidence of CAA in untreated patients, especially in the era of pre-intravenous immunoglobulin (IVIG), has been reported to be as high as 25%, though it is significantly reduced to 4%–10% in patients who receive timely administration of IVIG (1–4). Coronary artery lesions (CAL) in children with KD (up to 80% of all cases), can be identified with an initial echocardiography, during the first week of illness (5)*.* Changes in CALs from the initial echocardiography have been documented to regress in up to 75% of the cases, however 4%–5% of cases progress (6). In particular, over 80% of patients with coronary artery ectasia were reported to have an improved outcome. Baseline measurements of coronary artery diameter are realistic predictors of their involvement during early follow-up (7, 8). A major adverse cardiac event (MACE) has been shown to be correlated to higher CAA Z-scores at diagnosis, the presence of giant CAA, and the lack of IVIG treatment (9–11). Surveillance of myocardial stress tests and regular echocardiographies are warranted in the presence of CAA.

In contrast to the presence of CAA, the timing of follow-up echocardiography in patients with KD who do not have CAA at the initial echocardiography (coronary artery Z-score < 2.5) is still controversial. Based on 2017 American Heart Association guidelines, serial echocardiography is recommended at baseline, at one to two weeks, and at four to six weeks after diagnosis to detect coronary artery abnormalities. In patients without coronary artery involvement, discharge from cardiology service may reasonably take place at 4–6 weeks after KD onset, though extended follow-up to 12 months may be considered (1). The 2020 Japanese guidelines recommend serial echocardiography at the time of treatment initiation, 1–2 weeks later, and at follow-up assessments at 1, 2, 6, 12 months, and 5 years or yearly until 5 years for patients with KD and normal coronary artery or transient dilatation (2). In a large retrospective study from Boston and San Diego, new abnormalities in coronary arteries were found to be rare (1.7%), which suggests that the six-week echocardiographic imaging may not be needed in patients with uncomplicated KD and coronary artery Z-scores < 2.0 in the first two weeks of illness (12). In a recent study from the Post-RAISE registry in Japan; however, the optimal duration of echocardiographic follow-up is suggested to be one month in patients with no initial CAA and no CAL at one month (13). Based on these findings, new CAAs are assumed to develop frequently from the second week and then peak at the fourth week of illness in patients with no initial CAL (11, 14).

In low-income countries, frequent echocardiographic assessments can lead to high costs or risks for sedated patients. In a retrospective trial of the cost-effectiveness of echocardiography in 1999, all children with KD were recommended to have an echocardiography at the time of diagnosis with a follow-up study four to eight weeks after the onset of fever (15). We hypothesized that coronary arteries in patients without initial CAA and that remained normal at eight-weeks follow-up did not progress to CAA or require further intervention. Considering the prior studies that were conducted in different settings and among different ethnic groups, we undertook this multicenter retrospective study of patients diagnosed with KD without initial CAA (Z-score < 2.5). Our aim is to evaluate changes in the coronary artery Z-scores and occurrences of major adverse cardiac events from the initial diagnosis, to two weeks, eight weeks, and at one year following diagnosis.

## Methods

The present study was a multicenter retrospective study using hospital databases from four cardiac centers in Thailand: (1) Faculty of Medicine Siriraj Hospital, Mahidol University, Bangkok; (2) Phrapokklao Hospital, Chanthaburi; (3) Nakhon Pathom Hospital, Nakorn Pathom; and (4) Chao Phraya Yommarat Hospital, Suphanburi, Thailand. Following approval from the institutional ethics committees of all institutes, patients who were diagnosed with KD without CAA between July 1, 2017 and December 31, 2020 were retrospectively reviewed. We restricted the analyses to patients who had no initial CAA, which was defined as having a coronary Z-score < 2.5 at the initial echocardiography (1). Patients were also required to have had a complete echocardiographic measurement of the proximal right coronary artery (RCA), the left main coronary artery (LCA), and the left anterior descending artery (LAD) at least twice: at baseline and at eight weeks from the diagnosis. The echocardiographic measurements that were recorded when possible at two weeks and at one year following diagnosis were in accordance with guidelines. Patients who had only a single echocardiography without any follow-up were considered as incomplete data, and patients with congenital heart disease that could lead to coronary dilation were excluded from the study. The requirement for informed consent from patients was waived and the process for protecting patient confidentiality was guaranteed. Permission for the study protocol to waive the informed consent process was approved by the Siriraj Institutional Review Board, Faculty of Medicine, Siriraj Hospital, Mahidol University [Study number 244/2,564 (EC4), COA No Si 309/2021], Phrapokklao Hospital Chanthaburi [COA No. 034/65], Nakhon Pathom Hospital [Record No 028/2021, COA No. 029/2021], and Chao Phraya Yommarat Hospital, Suphanburi, Thailand [COA No. YM027/2565]. Demographic, clinical, initial laboratory, and echocardiographic findings of RCA, LCA, LAD, and treatment were explored. Demographic data was collected for gender, age at diagnosis of KD, and clinical presentation. The laboratory data included erythrocyte sedimentation rate (ESR), C-reactive protein (CRP), hematocrit (Hct), white blood cell (WBC), and platelet count. The echocardiographic findings were collected at the time of diagnosis, and at two weeks, eight weeks, and at one year following diagnosis. The dimensions of the RCA, LCA and LAD with their Z-scores were recorded (7). The left circumflex artery (LCx) was reported as an absolute number and not evaluated in the analysis due to its anatomical variability and the difficulty in obtaining images. Normal coronary dimension was defined as coronary Z-score < 2.0 and coronary ectasia was defined as coronary Z-score 2.0–2.49. Small CAA was defined as coronary artery Z-score ≥2.5 and <5 (1). Treatment of KD included receiving IVIG, onset of fever that received IVIG (within 10 days or after 10 days), requirement for retreatment with IVIG, and receiving adjunctive anti-inflammation medications. The primary outcome was a maximal coronary artery Z-score at follow-up echocardiography at eight weeks and at one year. The secondary outcomes were clinical data and the report of MACE at a one-year follow-up. MACE was identified if the patients had a cardiovascular-related illness including total coronary artery occlusion, congestive heart failure, clinical or imaging evidence of myocardial ischemia (MI), the requirement for coronary artery bypass grafting (CABG), or percutaneous coronary intervention (PCI) (9).

### Statistical methods

Statistical analyses were performed with SPSS 20.0 for Windows (SPSS Inc., Chicago, IL, United States). Demographic, clinical, laboratory, cardiac imaging, and KD treatment data were presented as frequencies with percentages for the categorical variables and mean ± SD or median with interquartile range for the continuous variables. The maximal coronary artery Z-score at initial, two weeks, eight weeks, and one year was expressed as median with interquartile range. Changes in the maximal coronary artery Z-score across the four time points were assessed using a non-parametric test (Friedman's test). Two-tailed *P* values were used, and values less than.05 were considered significant.

## Results

### Patient characteristics

**Figure 1:**
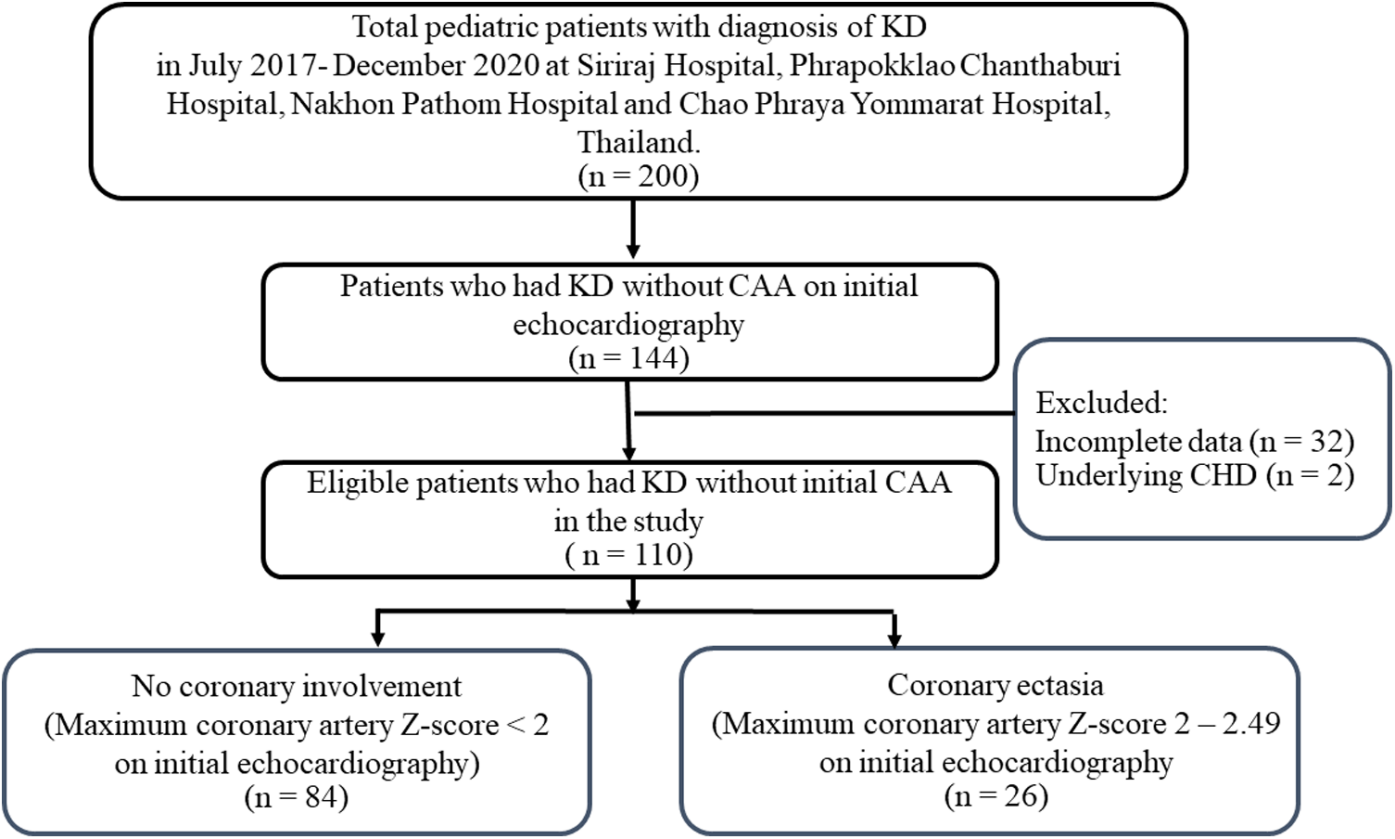
Flow diagram of pediatric patients that were included in the analysis (*n* = 110). KD, Kawasaki disease; CAA, coronary artery aneurysm.

A total of 200 patients had been diagnosed with KD in the 4 centers in 2017–2020. CAAs were noted in 56 patients (28%). Of the 144 patients without CAA, 33 patients had incomplete echocardiographic examinations and 1 patient had underlying congenital heart defect. Therefore, 110 patients were included in the study (Figure 1). Of these patients, 60% were male and the median age at diagnosis was 1.9 years (IQR 2 months, 3.2 years). Complete KD was diagnosed in 54.5% of the patients. No patients in this study had Kawasaki shock or ventricular dysfunction. Initial left ventricular ejection fraction was 68.4 ± 6.3%. Almost all of patients (90%) received IVIG as a standard treatment. IVIG-resistant KD presented in four patients, which responded to a second IVIG treatment plus steroids. Initial coronary artery Z-scores were normal (Z-score < 2) in 84 patients (76.4%). Of the remaining 26 patients, the maximal Z-score ranged from 2 to 2.47, which was consistent to coronary ectasia on initial echocardiography. The patients' demographics including clinical and laboratory data are described in Table 1.

**Table 1:** Baseline characteristics.

Variables	Total (*n* = 110)
Age at diagnosis (months)	23 (2, 39)
Weight at diagnosis (kg)	12.23 ± 5.01
BMI	16.10 ± 2.24
Male sex	66 (60%)
Incomplete KD	50 (45.5%)
Lack of IVIG treatment	4 (3.6%)
Timing of IVIG treatment	
≤10 days of fever	99 (90%)
>10 days of fever	7 (6.3%)
Onset of fever received IVIG (day)	6.60 ± 2.10
Retreatment with second IVIG	4 (3.6%)
Receiving adjunctive anti-inflammatory medication	4 (3.6%)
Hct (%)	30.99 ± 3.72
WBC (/mm^3^)	17,064 ± 5,249
Platelet (/mm^3^)	44,0411 ± 14,9018
ESR (mm/hr)	72.60 ± 29.28
CRP (mg/l)	111 ± 92
ALT	76 ± 107
Albumin	3.50 ± 0.51
Presence of sterile pyuria	12 (10.9%)
Initial Z-score of coronary artery	
RCA	0.81 (0.02, 1.64)
LCA	0.40 (−0.12, 1.14)
LAD	0.71 (−0.38, 1.40)
Presence of initial coronary ectasia (Z-score 2–2.49)	26 (23.6%)

Data presented as *n* (%), mean ± SD, and median (interquartile range).

KD, Kawasaki disease; IVIG, intravenous immunoglobulin; LCA, left main coronary artery; LAD, left anterior descending artery; RCA, right coronary artery; WBC, white blood cell; ESR, erythrocyte sedimentation rate; CRP, C-reactive protein; ALT, alanine aminotransferase.

### Changes in the coronary artery on echocardiography and clinical outcomes

The echocardiographic measurements were performed at baseline and at 8 weeks following diagnosis in 110 patients, and used as the entry criteria. Overall, serial echocardiographic examinations were performed in 64 patients (58%) at 2.7 weeks (IQR 2.1, 3.2 weeks), in 110 patients (100%) at 8.1 weeks (IQR 7.2, 8.8 weeks), and in 51 patients (46.3%) at 1 year (IQR 0.7, 1.3 years) (Table 2). The median Z-score of all coronary arteries tended to decrease over time. At the 2-week echocardiography (*n* = 64), progressive changes were seen in coronary arteries with small CAA, and Z-scores between 2.6 and 4.28 were seen for the first time in 4 patients (6.2%). Two patients were noted as having initial coronary ectasia and two patients had normal coronary artery Z-scores at their baseline echocardiography. No additional antithrombotic treatment was given. Three patients had dilatation of the coronary artery that progressed from normal to ectasia. Two patients with initial ectasia of the coronary artery had mildly dilated coronary arteries that did not change. In contrast, regression of coronary ectasia to normal occurred in nine patients (14%).

**Table 2:** Echocardiographic findings and clinical data at each time point.

	Initial (*N* = 110)	2 weeks (*N* = 64)	4–8 weeks (*N* = 110)	1 year (*N* = 51)
**Coronary artery Z-score**				
RCA	0.81 (0.02, 1.64)	−0.11 (−0.80, 0.43)	−0.03 (−0.51, 0.51)	−0.10 (−0.76, 0.33)
LCA	0.40 (−1.23, 1.14)	−0.18 (−0.77, 0.31)	−0.20 (−0.74, 0.32)	−0.30 (−1.00, 0.16)
LAD	0.71 (−0.18, 1.40)	−0.53 (−1.21, 0.31)	−0.29 (−1.01–0.43)	−0.54 (−1.30, 0.31)
**Maximal Coronary artery Z-score**	2.47	4.28	2.00	1.90
**Absolute number of coronary dimension (mm)**				
RCA	2.00 (1.70, 2.21)	1.71 (1.41, 2.00)	1.80 (1.53, 1.94)	1.83 (1.57, 2.07)
LCA	2.30 (2.10, 2.60)	2.20 (1.80, 2.46)	2.10 (1.90–2.40)	2.20 (2.00, 2.40)
LAD	1.80 (1.60, 2.00)	1.70 (1.31, 1.90)	1.7 (1.50, 1.80)	1.60 (1.50, 1.90)
**Presence of coronary ectasia**	26 (23.6%)	5 (7.8%)	1 (0.9%)	0
**Presence of coronary aneurysm**	0	4 (6.2%)	0	0
**MACE report**	0	0	0	0
**Functional class I**		64 (100%)	110 (100%)	90 (100%)*

Data is shown as *n* (%), median (interquartile range).

* Number of patients who had clinical follow-up at 1 year, *N* = 90.

LCA, left main coronary artery; LAD, left anterior descending artery; RCA, right coronary artery; MACE, major adverse cardiac events.

Of the 110 echocardiographic evaluations at 8 weeks, 109 patients (99.1%) showed normal coronary artery Z-scores (<2). Only one patient (0.9%) who had initial coronary ectasia had persistent coronary ectasia with a maximal Z-score of 2.0 (Table 3). Overall, the left ventricular ejection fraction was preserved at 68.5 ± 6.1%. All four of the small CAA that were seen at two weeks, had regressed to normal at the eight-week follow-up. Moreover, 25 (96.1%) of the 26 patients with initial coronary ectasia regressed to normal within 4–8 weeks of illness. The remaining patient with initial coronary ectasia, that had an unchanged risk level at eight weeks, resolved to normal at the one-year echocardiography. Of the 51 available one-year echocardiographies, all patients had coronary artery Z-score <2. The one-year clinical follow-up was conducted for 90 patients (81.8%). No MACE or mortalities were reported in this cohort study.

**Table 3:** Maximal coronary artery Z-scores of the left main coronary artery, left anterior descending artery, or right coronary artery at the 8-week echocardiography, compared to baseline, 2-week, and 1-year.

Maximal coronary artery Z-score	Number of patients	Maximal coronary artery Z-score at 8-week echocardiography		
Baseline	110	<2	2–2.49	2.5–5
<2	84	84 (100%)	0	0
2–2.49	26	25 (96.2%)	1 (3.8%)	0
2-week echocardiography	64			
<2	55	55 (100%)	0	0
2–2.49	5	5 (100%)	0	0
2.5–5	4	4 (100%)	0	0
1-year echocardiography	51			
<2	51	50 (98%)	1 (2%)	0

Data is shown as *n* (% in the row).

**Figure 2:**
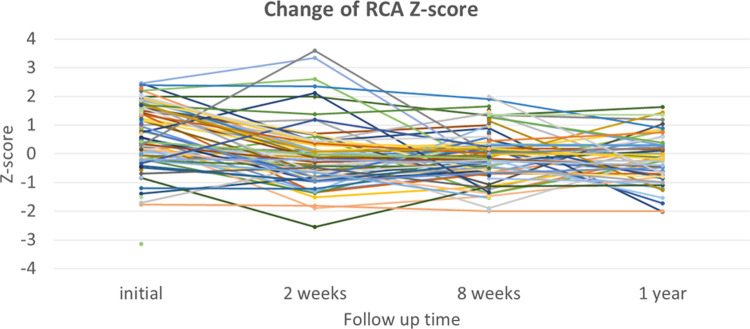
Changes in Z-scores for the right coronary artery (RCA) at each time point.

**Figure 3:**
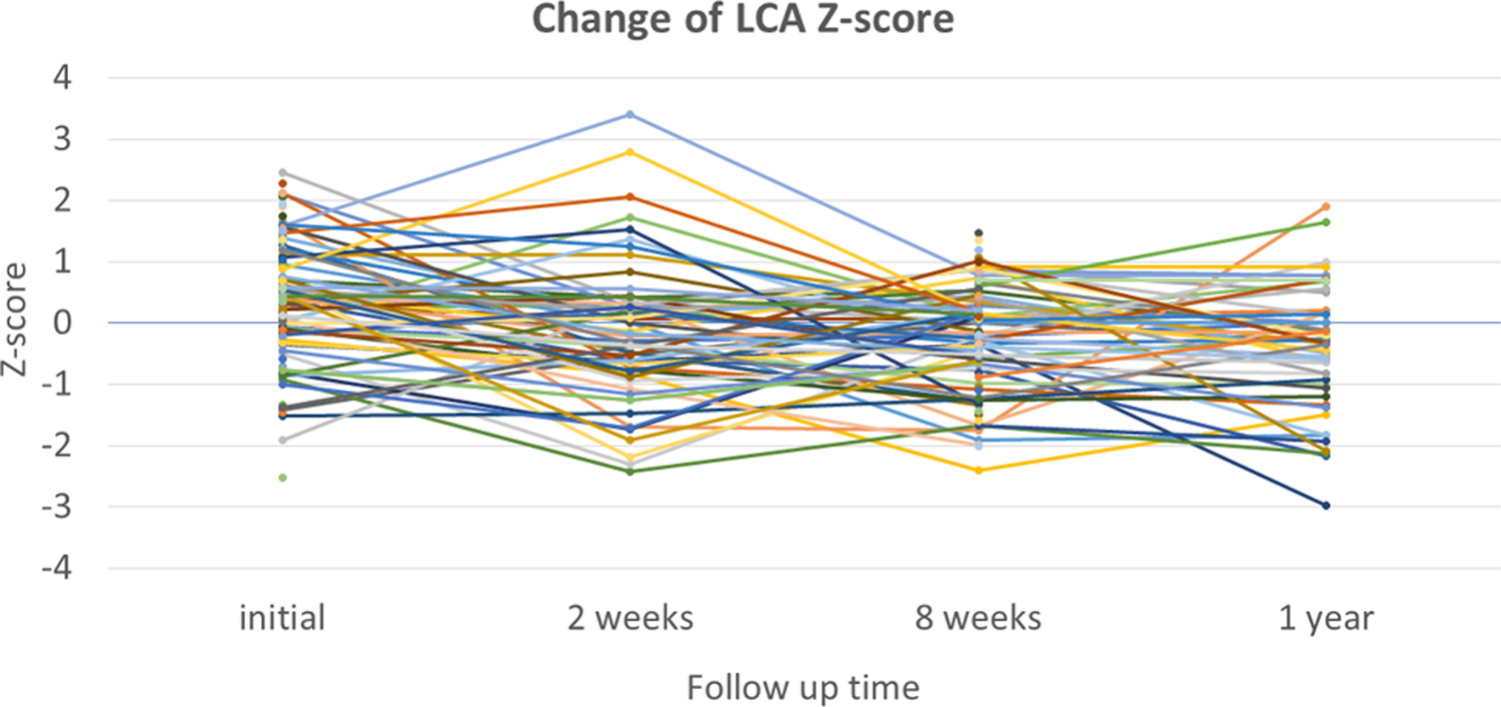
Changes in Z-scores for the left main coronary artery (LCA) at each time point.

**Figure 4:**
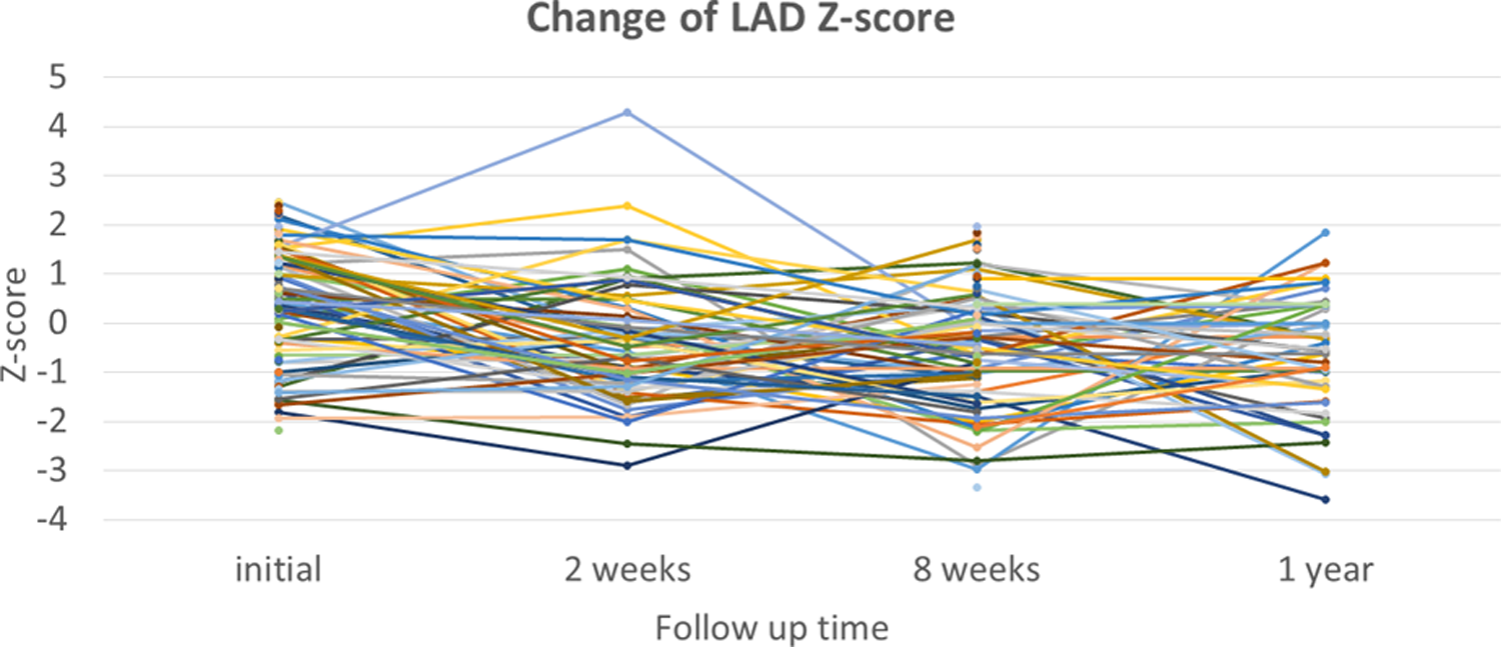
Changes in Z-scores for the left anterior descending artery (LAD) at each time point.

In the analysis of 55 patients who had normal coronary artery Z-scores at baseline and at 2 weeks, no patient had progressive dilation of the coronary artery to either ectasia or CAA at the 8-week echocardiographic examination (Table 3). Of the 55 patients, 48 patients visited a clinic and were asymptomatic at 1 year, and still showed a maximal coronary artery Z-score <2. Of the 84 patients who had normal coronary artery Z-scores at baseline and at 8 weeks, 37 patients had a fourth echocardiographic evaluation at 1 year that showed a normal coronary appearance. All patients with coronary ectasia or CAA at two weeks or at eight weeks underwent an echocardiography at the one-year follow-up, and showed resolution of all coronary lesions. If we consider the patients with coronary artery Z-score < 2 at two weeks or at eight weeks, who underwent an echocardiography at one year (*n* = 30 and *n* = 50, respectively), no evidence was found of worsening coronary dilatation at one year. These findings imply that progressive dilation of the coronary artery in patients who had been diagnosed with KD without CAA was rare, though it may have occurred at two to four weeks after illness. For most patients with dilation that included CAA and ectasia, their cases were benign and spontaneously resolved within four to eight weeks. No additional antithrombotic agents were given to this cohort. Patients who had normal coronary artery *Z* scores at their follow-up echocardiographies at two weeks or eight weeks continued to have normal *Z* scores at one year. Changes in the coronary artery Z-scores for RCA, LCA, and LAD for each patient are shown in Figure 2, Figures 3, 4, respectively.

Comparing patients with complete KD (*n* = 60) and incomplete KD (*n* = 50), the proportion of initial coronary ectasia was not statistically different (30% vs. 16%, *p* = 0.08). In the complete KD group (*n* = 60), 18 patients had initial coronary ectasia (30%), which regressed to a normal size in 17 patients (94%) at 8 weeks (Table 4). One patient who had residual coronary ectasia with a Z-score of two at eight weeks was resolved to normal at one-year follow-up (Table 4). Likewise, in the incomplete KD group (*n* = 50), initial coronary ectasia was noted in 8 patients (16%). All patients were resolved to normal at the eight-week echocardiography (Table 5). New findings of coronary aneurysms were detected by two-week echocardiography in three patients who had complete KD and in one patient who had incomplete KD. All coronary aneurysms were resolved to normal at eight-week and one-year echocardiography (Tables 4, 5). No cardiac events were reported.

**Table 4:** Maximal coronary artery Z-scores of the left main coronary artery, left anterior descending artery, or right coronary artery at the 8-week echocardiography, compared to baseline, 2-week, and 1-year of 60 patients with complete KD.

Maximal coronary artery Z-score	Number of patients	Maximal coronary artery Z-score at 8-week echocardiography		
Baseline	60	<2	2–2.49	2.5–5
<2	42	42 (100%)	0	0
2–2.49	18	17 (94.4%)	1 (5.6%)	0
2-week echocardiography	31			
<2	26	26 (100%)	0	0
2–2.49	2	2 (100%)	0	0
2.5–5	3	3 (100%)	0	0
1-year echocardiography	24			
<2	24	24 (100%)	0	0

Data is shown as *n* (% in the row).

**Table 5:** Maximal coronary artery Z-scores of the left main coronary artery, left anterior descending artery, or right coronary artery at the 8-week echocardiography, compared to baseline, 2-week, and 1-year of 50 patients with incomplete form of KD.

Maximal coronary artery Z-score	Number of patients	Maximal coronary artery Z-score at 8-week echocardiography		
Baseline	50	<2	2–2.49	2.5–5
<2	42	42 (100%)	0	0
2–2.49	8	8 (100%)	0	0
2-week echocardiography	33			
<2	29	29 (100%)	0	0
2–2.49	3	3 (100%)	0	0
2.5–5	1	1 (100%)	0	0
1-year echocardiography	27			
<2	27	27 (100%)	0	0

Data is shown as *n* (% in the row).

## Discussion

This multicenter retrospective study describes changes in coronary artery Z-scores in 110 children who were diagnosed with KD without CAA (Z-score < 2.5) at the initial management. Eighty-four patients (76.4%) had initial coronary artery Z-scores < 2 and 26 patients had initial coronary ectasia with a maximal Z-score of 2–2.47. At the eight-week follow-up echocardiography, only one patient (0.9%) with initial coronary ectasia had persistent coronary ectasia with a maximal Z-score of 2.0. All four patients with small CAAs that were detected at the two-week echocardiographic follow-up were resolved to normal in the eight-week examination. Of the 51 patients who received a one-year echocardiographic evaluation, all patients had coronary artery Z-scores < 2, including 1 patient who had persistent coronary ectasia at the 8-week echocardiography. Patients who had normal coronary artery Z-scores at the two-week or eight-week follow-up echocardiography continued to be normal at one year. In clinical follow-ups at one year (*n* = 90; 81.8%), no MACE or mortality were reported in this cohort study. We proposed that the optimal timing of echocardiographic follow-up could be two to eight weeks in patients without initial CAA and who still had a normal coronary appearance (coronary artery Z-score < 2) at their second echocardiographic evaluation. This suggested timing for the echocardiographic examination in this group would reduce costs, parental anxiety, and risks of sedation to patients for at least two evaluations, which would be suitable for low-socioeconomic countries.

Most coronary abnormalities in children with KD can be identified at the initial echocardiography, during the first week of illness (5). Coronary dilations, as a consequence of inflammation, usually peak around two to four weeks after illness onset (14). Patients with persistent CAA, defined as a *Z* score ≥ 2.5 after six weeks are considered to be at least risk level 3, which requires long-term management and lifetime specialist management from cardiology services based on whether or not assessing coronary insufficiency is required (1, 2, 16). In contrast, the cohort study of 258 children initially found to have normal coronary arteries at the first coronary angiogram, who were followed for 10–21 years, did not show any cardiac symptoms at follow-up (3). Hence, patients who have KD without CAA, or are at risk level 1 or 2 are widely accepted to have follow-ups by their general practitioner (1, 16). The recent 2017 AHA guideline recommends that assessment for coronary artery involvement in patients with KD is by serial transthoracic echocardiography, at diagnosis, and at two weeks and six weeks following onset of the disease, as a minimum. If abnormal, more frequent echocardiographies would be required to identify rapidly progressing CAA. In patients without coronary artery involvement, discharge from cardiology service would reasonably be at four to six weeks, but a follow-up at one year can be considered (1). The consensus recommendations from a UK group are similar to those of the AHA, with the recommendation of an additional echocardiographic evaluation at six months (16). Recent Japanese guidelines recommend the most frequent follow-up echocardiographies in patients without CAA to be at baseline, and at 1–2 weeks, 4 weeks, 8 weeks, 6 months, 12 months, and 5 years or yearly until 5 years (2). Undoubtably, frequent investigations would aid in the detection of new lesions, though the low incidence of new progressive coronary lesions and the needed therapy for this group should be weighed against the high cost of testing, the family's travel burden, and the added risk of sedation for most young children requiring echocardiographic evaluation.

The present study demonstrates that new CAA in-patients with KD with no previous CAA in their initial echocardiography are rare and all cases were resolved within eight weeks after illness without additional treatment. Furthermore, patients with normal echocardiographies at two weeks or eight weeks continued to be normal at one year, which implies the benign nature. Therefore, a second echocardiography may produce a high yield at two to eight weeks, and if the results show normal coronary arteries, further echocardiographic follow-up may not be indicated. This finding is comparable to prior reports (12, 13, 15). For example, a retrospective study of 67 patients with KD had follow-ups at 2–8 weeks and one patient had another follow-up. Scott et al. (15) found that 50 patients who had normal echocardiograms at 2–8 weeks after the onset of KD, continued to have normal coronary arteries at later follow-ups. Echocardiographic imaging beyond six to eight weeks appears to have little benefit (15). A recent report in Japan by Wang et al. (13) considered 386 children with KD who had no CAA at their baseline and had follow-ups at 1, 2, 4, and 8 weeks, and 1 year and 5 years. Nine patients (2.3%) were found to have new CAAs at one month with three of the nine patients (0.8%) having moderate CAA that required additional antithrombotic therapy. The remaining six of nine patients had coronary ectasia, which were resolved spontaneously at the subsequent follow-up. After four weeks, seven of nine patients developed new coronary artery lesions (Z-score > 2), three developed new lesions at eight weeks, and four developed new lesions at one year. In any case, their lesions were spontaneously resolved later. The authors concluded that the optimal timing for echocardiographic follow-up may be one month in patients with no initial CAA and coronary artery Z-scores < 2 at one month (13). de Ferranti et al. (12) reviewed a large database of 464 patients with KD who had coronary artery Z-scores < 2 at baseline and at 2 weeks following diagnosis. Their 6-week echocardiographic follow-ups showed that 456 patients (98.3%) continued to have normal Z-scores. Of the remaining eight patients (1.7%), five had coronary ectasia and three had CAAs. All coronary artery lesions subsequently regressed to normal. In this study, the authors suggested that the six-week echocardiographic imaging may be unnecessary in patients with uncomplicated KD and *Z* scores < 2.0 in the first two weeks of illness (12). From our study, and the above three studies, the follow-up timing of echocardiography differed slightly, but all of these studies provide evidence for a low incidence of new CAA in uncomplicated KD after the second echocardiography between two and eight weeks. All new cases of CAA tend to regress over time without the need for additional therapy. Therefore, we propose that subsequent echocardiographies in patients with KD who had no initial CAA and normal Z-scores at their second echocardiography that had been performed at two to eight weeks may not be necessary. In our study, a one-year clinical follow-up showed a good functional class without adverse cardiac symptoms, as was reported by previous authors (3, 12). In a study of 166 adults with a history of KD, all patients without CAA had normal coronary calcium scores, though significantly increased calcium scores were found in the patients with CAA (17). Thus, patients with KD and no CAAs can be classified into a low cardiovascular risk group, without needing to have long-term cardiology testing and stress imaging surveillance. Nevertheless, general counseling about a healthy lifestyle and activity is recommended for these patients (1, 16).

Our study had several limitations in its retrospective nature, with selection bias, small sample size, significant loss to follow-up at two-weeks and at one year, and possible measurement bias due to the use of multicenter data. In any case, all investigators were certified cardiologists and trained at the same institute (Siriraj Hospital); thus, we expected the methods of measurement to have only a small deviation. Z-scores were calculated using the same standard reference (7). Because the entry criteria were patients without CAA who had complete baseline and follow-up echocardiography at eight weeks, the rate of no echocardiographic follow-up at two weeks was 41%, which could underpower the actual rates of new coronary artery lesions. Nonetheless, the results of eight-week follow-up were complete and clinically meaningful. The clinical follow-up at 1 year occurred with 81.8% of the patients and the echocardiographic follow-up occurred with 46.3% of the patients. Again, the results at the one-year follow-up could be an underestimation. Consequently, we analyzed the data carefully and identified the number of patients at each visit (Table 3).

## Conclusion

New CAA in patients with KD who had no previous CAA in their initial echocardiography are rare. The patients who had a normal echocardiographic follow-up at two weeks or eight weeks mostly continued to have normal assessments at one year. The optimal timing for the echocardiographic follow-up should be at two to eight weeks in patients without initial CAA and still having a coronary artery Z-score < 2 in the second echocardiographic examination. While clinical follow-ups at one year may be considered, the decision should involve the physician and family counsellor.

## Data Availability

The raw data supporting the conclusions of this article will be made available by the authors, without undue reservation.
